# Genome-Wide Identification and Expression Pattern Analysis of the *GATA* Gene Family Members in *Scutellaria baicalensis* Georgi Under Carbon Dot Treatment

**DOI:** 10.3390/biology15110834

**Published:** 2026-05-26

**Authors:** Qingbo Meng, Junbai Ma, Meitong Pan, Lingyang Kong, Qingdong Gao, Weichao Ren, Wei Ma, Xiubo Liu

**Affiliations:** 1 College of Pharmacy, Heilongjiang University of Chinese Medicine, Harbin 150040, China; mengqingbo95622@163.com (Q.M.); 15114516116@163.com (J.M.); meitong_pan@163.com (M.P.); hljkly970219@163.com (L.K.); gaoqingdong123456@163.com (Q.G.); renweichao@hljucm.edu.cn (W.R.); 2 School of Chinese Medicine, Hong Kong Baptist University, Hong Kong, China; 3 College of Jiamusi, Heilongjiang University of Chinese Medicine, Jiamusi 154007, China

**Keywords:** *Scutellaria baicalensis* Georgi, carbon dots, *GATA* gene family, chlorophyll

## Abstract

*Scutellaria baicalensis* Georgi is a crop with significant economic and medicinal value, but no studies have examined the *GATA* gene family in *S. baicalensis* or its expression patterns following foliar spray with nanomaterials. In this study, the *GATA* gene family in *S. baicalensis* was identified and analyzed using bioinformatics. The results showed that 25 *SbGATAs* genes were identified, distributed across seven chromosomes, and could be classified into four subfamilies based on phylogenetic analysis. The physicochemical properties of SbGATAs proteins showed differences in the number of amino acids, molecular weight, and isoelectric point, but all were hydrophilic nuclear proteins. Analysis of cis-acting elements in the promoter regions revealed that the *SbGATAs* gene promoters were enriched for light, hormone response, and stress response elements. In this study, RT-qPCR was used to investigate the expression patterns of the *SbGATAs* family members in *S. baicalensis* leaves sprayed with different concentrations of carbon dot solutions. This study employed the RT-qPCR method to elucidate the expression of members of the *SbGATAs* family in the leaves of *S. baicalensis* after different treatments. The results showed that at low concentrations, except for *SbGATA6*, whose expression was upregulated by 2.1 times, the expressions of the other four genes were all downregulated; at high concentrations—except for *SbGATA6*, whose expression was upregulated by 3.75 times, and *SbGATA14*, the expression of which was basically the same as that of the control—the expressions of the other three genes were all downregulated. *SbGATA6* was the only gene that was significantly upregulated at both concentrations and whose upregulation ratio increased with the increase in concentration, indicating that it may have a wide response to carbon dot treatment and may be involved in physiological regulation at different concentrations.

## Simple Summary

The accumulation of active components in the roots of *Scutellaria baicalensis* Georgi, a major traditional Chinese medicinal herb, is closely related to the photosynthesis of its leaves and the metabolism of chlorophyll. This study systematically identified 25 members of the *GATA* gene family in *S. baicalensis* and analyzed the potential role of *SbGATA6* under carbon dot treatment. These findings provide important clues for understanding the molecular mechanism by which carbon dots regulate the chlorophyll accumulation and photosynthetic efficiency of *S. baicalensis*.

## 1. Introduction

*Scutellaria baicalensis* Georgi, a perennial herb of the genus Scutellaria in the *Lamiaceae* family, is used in traditional Chinese medicine, where the dried underground parts (roots) serve as the medicinal material. It is usually harvested in spring and autumn. The use of this type of medicinal material was first recorded in the ShenNongBenCaoJing (the earliest existing traditional Chinese medicine book), and it has been widely used in traditional medicine for thousands of years. It exhibits heat-clearing, dampness-drying, fire-purging, detoxifying, hemostatic, and fetus-stabilizing properties. Modern pharmacological studies have shown that it possesses a variety of pharmacological activities, including anti-inflammatory, antiviral, anti-tumor, antioxidant, lipid-lowering, and hypoglycemic effects [1,2,3,4,5]. In recent years, due to its excellent therapeutic effect and the excessive exploitation of wild resources of *S. baicalensis*, it has been facing depletion. Artificial cultivation is currently the main source of *S. baicalensis*, but it is confronted with multiple problems, such as long-term monoculture and soil degradation, which weaken crop adaptability, reduce yield, and lower the content of medicinal components. At the same time, excessive use of fertilizers and pesticides not only disrupts the soil microbial balance but may also pose safety risks, such as from heavy metal residues, further threatening the quality of medicinal plants and the ecological environment. Currently, in cultivation, traditional fertilizers are mainly used. Although traditional fertilizers can increase crop yield, they often come with problems such as environmental pollution and resource waste. Therefore, developing an efficient, green new type of fertilizer has become the focus of research. The emergence of nano-fertilizers is a direct response to this challenge. Compared with traditional fertilizers and pesticides, nano-fertilizers are more efficient, environmentally friendly, and can enhance crop growth and resilience, thereby reducing the environmental impact of agriculture [6]. Carbon dots (CDs), as a highly promising agricultural nanomaterial, offer a more environmentally friendly preparation and application process that meets the requirements of sustainable agriculture. It provides a new technical path to achieving more efficient, environmentally friendly “smart fertilizers” or “functional fertilizers” and is currently a research hotspot in green agricultural technology. Carbon dots stand out in this environment.

Carbon dots are an emerging class of carbon-based nanomaterials. The earliest discovery of this material dates back to 2004 [7], but it was not until 2006 that these fluorescent nanoparticles were officially named carbon dots [8]. CDs have attracted significant attention due to their unique advantages, such as extremely small size (<10 nm), distinctive fluorescence properties, low toxicity, good water solubility, excellent biocompatibility, and environmental friendliness [9]. Currently, they have broad application potential in various fields such as sensing, catalysis, biomedicine, and drug delivery [10,11,12,13]. In 2012, it was first demonstrated that CDs have excellent biocompatibility with plants [14], and more and more studies have focused on the effects of CDs on the growth of various plants and their mechanisms of action. To date, CDs have demonstrated superior performance in regulating various plant physiological processes, including growth and development, photosynthesis, and resistance to biological and non-biological stress [15,16,17]. *S. baicalensis*, a traditional major medicinal herb in China, has the accumulation of active components in its roots closely related to the photosynthesis and chlorophyll metabolism of its leaves. However, the specific molecular mechanisms by which carbon dots regulate plant photosynthesis remain unclear. Given that the expression of photosynthesis-related genes is often precisely controlled by specific transcription factors, carbon dots may mediate their photosynthetic-promoting effects by influencing certain transcription factor families. Multiple studies have shown that GATA transcription factors play important roles in plant growth and development, photosynthesis, responses to biotic and abiotic stresses, and hormone signal transduction [18,19,20,21,22]. However, the functions and characteristics of the *GATA* gene family in *S. baicalensis* remain unexplored. Based on this, we propose the hypothesis that carbon dots may influence chlorophyll metabolism and photosynthetic efficiency by modulating the expression of specific members of the GATA family in *S. baicalensis*.

The GATA transcription factors are widely present in eukaryotic organisms such as fungi, animals, and plants, and are named for their ability to specifically recognize and bind to the W-GATA-R sequence in the promoter region of target genes (W = T/A, R = G/A) [23]. GATA proteins all contain a highly conserved type IV zinc finger motif (C-X2-C-X17-20-C-X2-C) [24,25]. Most plant GATA transcription factors contain only a single zinc finger domain, while a few contain two zinc finger domains [25]. The biological functions of GATA factors in plants have been extensively studied. Since the first plant GATA transcription factor, NTL1, was isolated from tobacco in 1993 [26], more and more GATA transcription factors have been extensively studied, such as *Arabidopsis thaliana* and *Oryza sativa* [25,27].

Therefore, this study aims to identify and comprehensively analyze the members of the *GATA* gene family in *S. baicalensis* based on its genome using bioinformatics techniques. By combining the expression changes under the treatment of novel nanomaterial carbon dots, it provides important clues for understanding the molecular mechanism of carbon dots in regulating chlorophyll accumulation and photosynthetic efficiency in *S. baicalensis*. This research offers a theoretical basis and genetic resources for optimizing the growth of medicinal plants, enhancing light energy utilization efficiency, and increasing the yield of active components through carbon dot nanotechnology. It has clear scientific significance and potential application value for molecular-assisted breeding and green and efficient cultivation of *S. baicalensis*.

## 2. Materials and Methods

### 2.1. Identification and Physicochemical Property Analysis of the GATA Gene Family in S. baicalensis

The genome and annotation files of *S. baicalensis* were obtained from the Database Resources of the National Genomics Data Center (Accession number: GWHDEDD00000000) [28,29]. Firstly, the Hidden Markov Model file (PF00320) containing the GATA conserved domain was downloaded from the Pfam database [30,31]. Using HMMER 3.0, the downloaded model file was used as a query to conduct an initial search for the *S. baicalensis GATA* gene across the entire genome [32]. Next, 30 *A. thaliana* GATA protein sequences were downloaded from the TAIR database, and a local BLAST v2.17.0 database was constructed [33]. The GATA proteins identified in the preliminary search were used in a BLAST 2.17.0 search of the NCBI database to obtain the final GATA protein sequence of *S. baicalensis*. The physicochemical properties of the candidate gene-encoding protein were analyzed using the ExPASy online tool [34]. The subcellular localization of the protein was predicted using the Plant-mPLoc 2.0 website [35].

### 2.2. Systematic Analysis of the GATA Gene Family Evolution in S. baicalensis and A. thaliana

A multiple sequence alignment of *A. thaliana* and 25 *S. baicalensis* GATA protein sequences was performed using MEGA11.0.13. Subsequently, a phylogenetic tree was constructed via the Neighbor-Joining method with 1000 bootstrap replicates and default parameters. The Newick file obtained was beautified for the evolutionary tree through the Evolview online website [36]. The *S. baicalensis GATA* gene family was classified according to the subfamily classification criteria of the *A. thaliana GATA* gene family.

### 2.3. Analysis of Chromosomal Localization of the GATA Gene Family in S. baicalensis and Intra-Species Collinearity Analysis

Chromosome location information for the *GATA* gene was extracted from the *S. baicalensis* annotation file using TBtools v.2.376, visualized, and subsequently the members of this gene family were named based on their positions on the chromosome [37].

Subsequently, the co-linear segments and gene duplication events existing in the genome were analyzed through the MCScan program algorithm of the TBtools software, in combination with the alignment results between sequences and the annotation files. The resulting collinear relationships were visualized with the Advanced Circos tool [37].

### 2.4. Characterization of Multiple Sequence Alignment, Conserved Motif Detection, and Gene Structure Analysis for the GATA Gene Family in S. baicalensis

Using MEGA11, we conducted a multiple sequence alignment of the amino acid sequence of 25 GATA protein derived from *S. baicalensis* and subsequently exported a FASTA file. The comparison results were visualized using the Multiple Alignment Trimming tool in TBtools [37].

Conserved motifs in the GATA proteins of *S. baicalensis* were annotated using MEME (maximum motif number set to 8, other parameters default) [38]. Conserved domains were analyzed with NCBI Batch CDD [39]. Exon–intron positions were obtained from *S. baicalensis* Gff3 annotations, and gene structures were visualized using TBtools and CFVisual v2.1.5 [37,40].

### 2.5. Promoter Region Cis-Element Analysis of the GATA Gene Family from S. baicalensis

The 2000 bp DNA sequences upstream of the start codon of *SbGATA* gene family members were extracted using the GXF Sequences Extract function in TBtools. The extracted sequences were then uploaded to the PlantCARE database for the prediction of cis-regulatory elements using default parameter settings [41]. Finally, the prediction results were visualized using the HeatMap function of TBtools, and the information was classified into responses to light, hormones, stress, and other responses.

### 2.6. Application of Carbon Dots on the Leaf Surface of S. baicalensis and Determination of Chlorophyll Content in S. baicalensis Leaves

Six-month-old *S. baicalensis* were transplanted into plastic pots filled with vermiculite and nutrient soil (1:1) and grown in a plant lighting incubator with 16 h of light and 8 h of darkness, with the temperature set at 25 °C. The research group previously prepared carbon dots via a hydrothermal method using citric acid and p-aminobenzenesulfonic acid as precursors. Different concentrations of treatment solutions (300 mmol/L and 500 mmol/L) were prepared and applied to the leaves of *S. baicalensis* for 30 days. The control group was distilled water. Plants were sprayed once a day, until the leaf surface was completely moist and no liquid dripped. Both the blank group and the treatment group had three biological replicates. The collected samples were quickly frozen with liquid nitrogen and stored in a −80 °C refrigerator.

The content of chlorophyll a and b in the *S. baicalensis* leaves was determined by spectrophotometry. The surface of the *S. baicalensis* leaves was wiped clean. A total of 0.1 g of the sample was weighed and cut it into a test tube containing 10 mL of 95% ethanol solution. The leaves were dark-treated for 24 h to fully release chlorophyll, and the measurement was carried out when the leaves in the test tube were completely white. The 95% ethanol solution was used as the control. The control and the extract were transferred to glass cuvettes, and the absorbance at 663 nm and 645 nm was measured, respectively. The total chlorophyll content in the leaves was calculated using the Arnon formula [42]. The formula is as follows:

Ca = (12.7A_663_ − 2.69A_645_)

Cb = (22.9A_645_ − 4.68A_663_)

C = Ca + Cb = (20.21A_645_ + 8.02A_663_)

Here, A_645_ and A_663_ represent the absorbance of the extract at 645 nm and 663 nm, respectively; Ca and Cb represent the content of chlorophyll a and chlorophyll b in the solution (unit: mg/L); C represents the content of chlorophyll in the solution (unit: mg/L). The content of chlorophyll in the solution needs to be converted into the content of chlorophyll in *S. baicalensis* leaves Chl (unit: mg/g) through the following formula:

Chl = V × C/1000 M

V represents the volume of the extract (in liters), and M represents the mass of the leaf (in milligrams).

### 2.7. Determination of Oxidative Stress and Antioxidant Indicators in S. baicalensis Leaves After Foliar Application of Carbon Dot Solution

The oxidative stress and antioxidant indicators of *S. baicalensis* leaves after foliar application of carbon dot solution were determined using the superoxide dismutase (SOD), catalase (CAT), peroxidase (POD) activity and malondialdehyde (MDA) content kits (Suzhou Grace Biotechnology Co., Ltd., Suzhou, China). A total of 0.1 g of the sample was added to an appropriate amount of extraction solution for ice bath homogenization. It was centrifuged at 12,000 rpm for 10 min at 4 °C, and the supernatant was taken for measurement. Each group of samples was measured according to the instructions of the kit, and technical repetition was performed three times for each group.

### 2.8. RNA Extraction, cDNA Synthesis, and qRT-PCR

RNA was isolated from *S. baicalensis* leaves using the Plant Total RNA Extraction Kit (Simgen Biotechnology Co., Ltd., Hangzhou, China). Subsequently, cDNA was obtained by reverse transcription using the reverse transcription kit (Nanjing Vazyme Biotech Co., Ltd. Nanjing, China). The expression profiles of the *SbGATA* gene family Class B subfamily in the leaves of *S. baicalensis* under two different concentrations of carbon dot solutions were detected by qRT-PCR. Primers were designed using the Primer3 web server [43]. *SbActin* was selected as the internal reference gene [44]. The experiment was repeated three times, and the relative gene expression levels were calculated using the 2^®−ΔΔCt^ method [45].

## 3. Results

### 3.1. Identification and Protein Characterization Analysis of the GATA Transcription Factor Family in S. baicalensis

**Figure 1:**
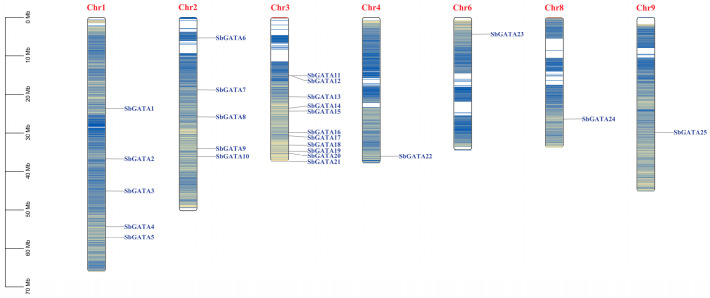
Distribution of *GATA* genes on chromosomes of *S. baicalensis*.

25 members of the *GATA* gene family were identified in the *S. baicalensis* genome (Table 1), distributed across seven chromosomes. Among them, Chr 3 contained the most *SbGATA* genes. Chr 1 and 2 each contained five *SbGATA* genes, while Chr 4, 6, 8, and 9 each contained one *SbGATA* gene (Figure 1). According to their chromosomal positions, the genes were designated *SbGATA1*-*SbGATA25*.

Using the ExPASy online platform, physicochemical analysis of *GATA* family members in *S. baicalensis* showed that the *SbGATA* encoded proteins ranged from 101 amino acids (SbGATA18) to 651 aa (SbGATA25) in length, with corresponding relative molecular weights of 11.6 kDa (SbGATA18) to 72.5 kDa (SbGATA25). The relatively short length of SbGATA proteins may indicate functional simplification or specificity. The theoretical pI ranged from 4.87 (SbGATA5) to 10.38 (SbGATA18), among which eight were basic proteins and the rest were acidic proteins. Most SbGATA proteins in the same group had similar theoretical pI values. For example, the theoretical pI values of all SbGATA proteins in group B were higher than 8, while all proteins in group C were lower than 8. The instability index of all SbGATA proteins was greater than 40. The aliphatic index ranged from 45.47 (SbGATA24) to 98.40 (SbGATA25). It is also worth noting that the average coefficient of hydrophobicity (GRAVY) of all SbGATA proteins was less than zero, and all 25 SbGATA proteins were hydrophilic proteins.

### 3.2. Multiple Sequence Alignment of the SbGATA Gene Family and Systematic Phylogenetic Analysis of the GATA Gene Family in S. baicalensis and A. thaliana

Based on the classification of GATA proteins in *A. thaliana*, the SbGATA proteins are divided into four subfamilies (Classes A–D). The Class A subfamily has the largest number of members, with 11: SbGATA1, SbGATA2, SbGATA4, SbGATA7, SbGATA10, SbGATA13, SbGATA16, SbGATA17, SbGATA20, SbGATA22, and SbGATA23. The Class B subfamily contains six members: SbGATA6, SbGATA8, SbGATA14, SbGATA18, SbGATA24, and SbGATA25. The Class C and Class D subfamilies each have four members. Class C includes SbGATA5, SbGATA9, SbGATA19, and SbGATA21. Class D consists of SbGATA3, SbGATA11, SbGATA12, and SbGATA15.

**Figure 2:**
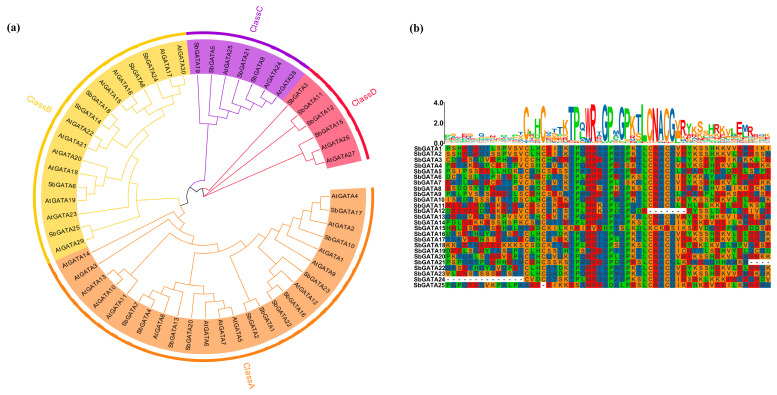
Phylogenetic analysis of the *GATA* gene family members in *A. thaliana* and *S. baicalensis* (**a**) and multiple sequence alignment of the *SbGATA* gene family (**b**).

The results of multiple sequence alignment indicate that most members of the *SbGATA* gene family contain a highly conserved zinc finger domain (C-X2-C-X18/20-C-X2-C), but the SbGATA12 and SbGATA25 domains show varying degrees of deletion (Figure 2). The GATA domains of the D subclass members (SbGATA15) are located near the 5′ end, while the GATA domains of other genes are all near the 3′ end. The conserved motifs of the domains of most members of the *S. baicalensis GATA* gene family are C-X2-C-X18-C-X2-C, while the domain motifs of the Class C subclass members are C-X2-C-X20-C-X2-C. These results indicate that the *S. baicalensis GATA* gene family as a whole has strong conservation, and the homology among the subfamilies is high. Most amino acid positions of the GATA zinc finger domains within and between subfamilies are highly similar.

### 3.3. Intraspecific Colinearity Analysis of the SbGATA Gene Family

**Figure 3:**
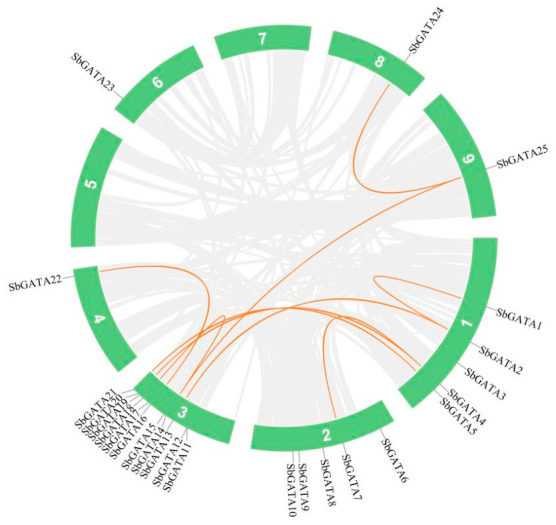
Intra-species collinearity analysis of the *SbGATA* gene family.

The relationship between the *SbGATAs* gene and gene duplication was investigated using intraspecific collinearity analysis (Figure 3). The results showed that no tandem duplications were found among the 25 *SbGATA* genes, and only nine segmental duplications were observed. They were *SbGATA1*/*SbGATA2*, *SbGATA2*/*SbGATA13*, *SbGATA4*/*SbGATA7*, *SbGATA4*/*SbGATA20*, *SbGATA5*/*SbGATA21*, *SbGATA14*/*SbGATA25*, *SbGATA14*/*SbGATA18*, *SbGATA16*/*SbGATA22*, and *SbGATA24*/*SbGATA25*. This suggests that segmental duplication events are the primary driver behind the expansion of this gene family.

### 3.4. Gene Structure and Conservation Motif Analysis of the SbGATA Gene Family

**Figure 4:**
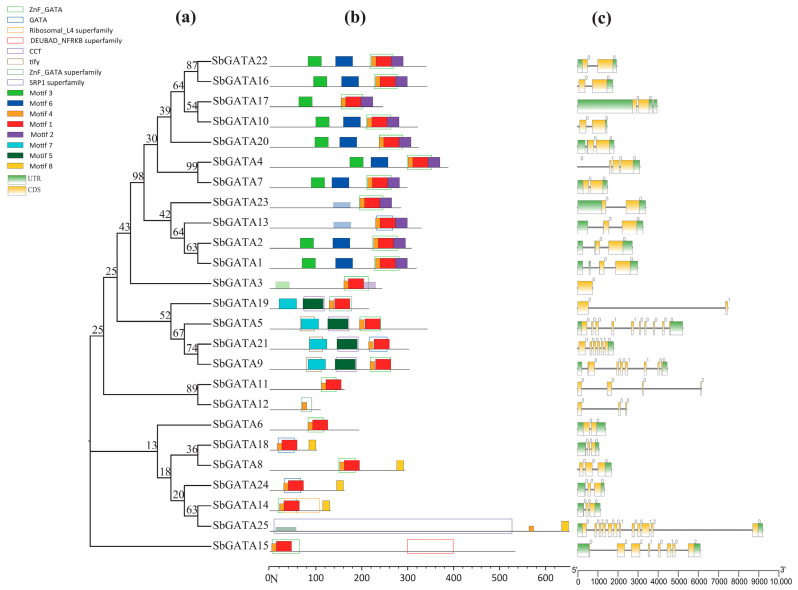
Sequence structure analysis of *SbGATA* gene family proteins. Note: (**a**) Systematic evolutionary tree of *SbGATA* gene family, (**b**) conserved motifs and conserved domains of proteins, (**c**) gene structure.

In this study, eight conserved motifs (Motif 1–8) were identified (Figure 4). All SbGATA proteins contain Motif 4. Members of the Class A subfamily all have three identical conserved motifs, namely Motif 1, 2, and 4, among which Motif 2 is only present in this subfamily. Members of the Class B subfamily all have one identical conserved motif, namely Motif 1. Members of the Class C subfamily all have four identical conserved motifs, namely Motif 1, 4, 5, and 7, among which Motif 5 and 7 are only present in this subfamily.

Exon–intron analysis revealed that seven members of this family have only one intron, 10 members have two introns, two members have three introns, one member has five introns, two members have seven introns, and one member has nine introns. Among them, SbGATA25 contains the most introns (*n* = 11).

### 3.5. Promoter Region Cis-Element Analysis of the SbGATA Gene Family

**Figure 5:**
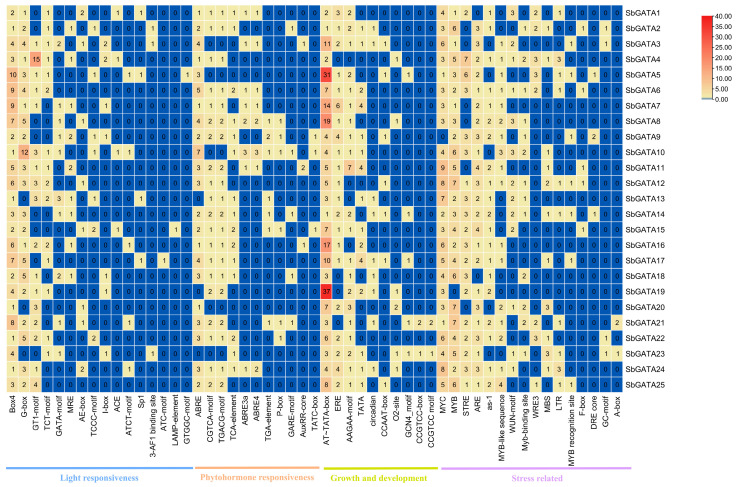
Analysis of cis-acting elements in the promoter of the *SbGATA* gene family.

A total of 25 categories of light-responsive cis-acting elements, including Box 4, G-box, and the GT1 motif, were present in the 25 *SbGATA* gene promoters (Figure 5). It is worth noting that the number of light-responsive cis-acting elements is the largest, with a total of 336.

The *SbGATA* gene promoter sequences were also found to contain many cis-acting elements involved in hormone (e.g., methyl jasmonate, salicylic acid, gibberellin, auxin) and abiotic and biotic (e.g., drought, low temperature) responses, along with elements related to meristem tissue expression and circadian rhythm control. These results indicate that members of the *SbGATA* gene family may be widely involved in various biological processes, including the growth and development of *S. baicalensis*, photosynthesis, responses to biotic and abiotic stresses, and hormone signal transduction, and may play a key role in these processes.

For example, *SbGATA2*, *SbGATA10*, *SbGATA14*, *SbGATA18,* and *SbGATA24* have GARE-motif; *SbGATA8*, *SbGATA9*, *SbGATA10*, *SbGATA15*, *SbGATA21*, and *SbGATA22* have P-box; *SbGATA9*, *SbGATA10*, and *SbGATA15* have TATC-box. GARE-motif, P-box, and TATC-box all belong to gibberellin response elements.

*SbGATA3*, *SbGATA11* (2), and *SbGATA16* have AuxRR-core; *SbGATA8*, *SbGATA9* (2), *SbGATA10*, *SbGATA13*, *SbGATA14*, *SbGATA15*, and *SbGATA21* have TGA-element. AuxRR-core and TGA-element both belong to auxin response elements.

### 3.6. Changes in Chlorophyll Content and Expression Patterns of the SbGATA Gene Under Foliar Application of Carbon Dot Materials

**Figure 6:**
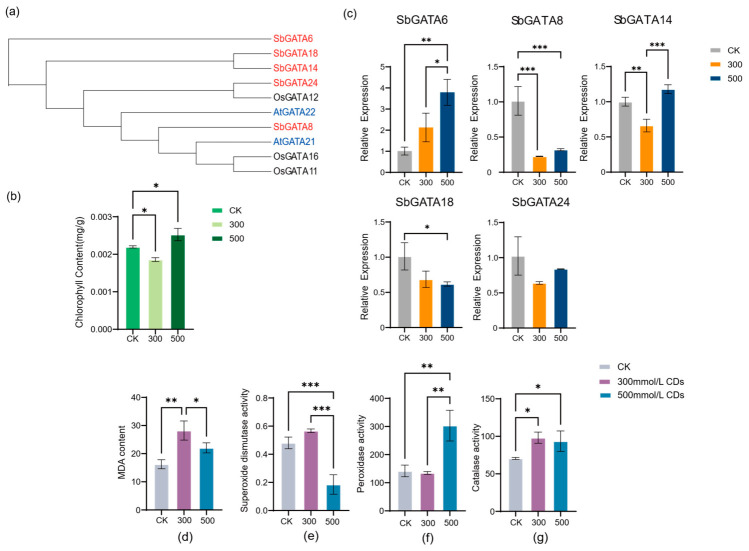
(**a**) Potential genes for chlorophyll biosynthesis regulation in the *GATA* gene family of *S. baicalensis.* (**b**) Changes in chlorophyll content under foliar application of carbon dot materials. (**c**) Expression of the *GATA* gene in *S. baicalensis* leaves after foliar application of carbon dot solutions at different concentrations. (**d**) MDA content. (**e**) Superoxide dismutase activity. (**f**) Peroxidase activity. (**g**) Catalase activity (* *p* < 0.05, ** *p* < 0.01, *** *p* < 0.001).

Through the research, it was found that after applying different concentrations of carbon dot solutions to the leaves of *S. baicalensis*, the results showed that the chlorophyll content in the leaves of *S. baicalensis* treated with 300 mmol/L concentration was lower than that of the blank group, while the chlorophyll content in the leaves treated with 500 mmol/L concentration was higher than that of the blank group (Figure 6). Under the treatment of 300 mmol/L carbon dots, the SOD activity in the *S. baicalensis* leaves significantly increased while the CAT and POD activities were relatively low. As a result, the H_2_O_2_ produced by the disproportionation of superoxide anions failed to be effectively eliminated, and the oxidative damage (indicated by the MDA content) was relatively severe, with the chlorophyll content decreasing. In contrast, under the 500 mmol/L treatment, although the SOD activity was relatively low, the CAT and POD activities significantly increased, and H_2_O_2_ was rapidly decomposed. The oxidative damage was relatively mild. Meanwhile, the phototransformation characteristics of the high-concentration carbon dots may directly promote photosynthesis, and the moderate H_2_O_2_ can act as a signaling molecule to activate the photosynthetic-related pathways, jointly leading to an increase in chlorophyll content. These results indicate that the concentration-dependent regulation of carbon dots on the chlorophyll metabolism of *S. baicalensis* is closely related to the induction of different oxidative stress patterns.

Through phylogenetic analysis, the *GATA* genes that may be involved in chlorophyll synthesis were also found to exhibit different expression patterns under the two spraying concentrations. The results showed that, at low concentrations, except for *SbGATA6*, the other four genes were down-regulated; at high concentrations, except for *SbGATA6* and *SbGATA14*, the other three genes were down-regulated. It is worth noting that at low concentrations, *SbGATA6* expression was up-regulated 2.1-fold, and at high concentrations, it was up-regulated 3.75-fold.

## 4. Discussion

### 4.1. Bioinformatics Analysis of the GATA Gene Family in S. baicalensis

The GATA transcription factors play a crucial regulatory role in various processes such as plant growth and development, photosynthesis, responses to biotic and abiotic stresses, and hormone signal transduction [18,19,20,21,22]. Currently, the *GATA* gene family has been identified in many plants. Compared to animals, there are more GATA proteins encoded in the plant genome, such as *A. thaliana* (30), *O. sativa* (28), *Dimocarpus longan* (22), *Citrus sinensis* (24), and *Hordeum vulgare* (27) [25,46,47,48]. This study is based on genome data from *S. baicalensis* and uses bioinformatics techniques to identify and analyze the physicochemical properties, gene structures, conserved domains, phylogeny, and promoter cis-acting elements of SbGATA family members. A total of 25 GATA gene family members were identified, unevenly distributed across seven chromosomes. In terms of quantity, it is more than *D. longan* and *C. sinensis*, and less than *A. thaliana*, *O. sativa*, and *H. vulgare*. These differences may be caused by the genome size and complexity among these species. The 25 SbGATA transcription factor proteins exhibit significant differences in molecular weight (Mw), suggesting that SbGATA transcription factors have undergone varying degrees of differentiation over long-term evolution to adapt to environmental changes. The theoretical pI ranges from 4.87 (SbGATA5) to 10.38 (SbGATA18), and most SbGATA proteins within the same group have similar theoretical pI values. Subcellular localization prediction indicates that all SbGATA proteins are located in the cell nucleus, suggesting that they play a dominant role in regulating transcription within the cell nucleus. Additionally, phylogenetic analysis reveals that the SbGATA family members can be divided into four subfamilies, consistent with the classification of *A. thaliana*. This further confirms the conservation of the *GATA* gene family in evolution. Multiple sequence alignment results show that most GATA amino acid sequences within a given subfamily exhibit similar structural characteristics. The zinc finger motif of the Class C subfamily is C-X2-C-X20-C-X2-C. According to previous studies, this GATA type may be an evolved subfamily with a relatively high degree of complexity that emerged after the differentiation of monocotyledons and dicotyledons [49]. Members of the *S. baicalensis* GATA gene family exhibit significant diversity in gene structure. The analysis of conserved domains shows that all SbGATAs proteins contain a highly conserved GATA zinc finger domain. However, the distribution of conserved motifs varies among different subfamily members, and this difference may be related to the functional diversity of the GATA genes. For example, Motifs 1, 2, and 4 are mainly concentrated in the members of the Class A subfamily, while Motifs 5 and 7 are mainly distributed in the Class C subfamily. Structural differences may affect the function of GATA genes across different biological processes. In terms of gene structure, the number and length of exons and introns of the SbGATA family members also show significant differences. The diversity of structure may be closely related to the complexity of post-transcriptional splicing, transcriptional regulatory mechanisms, and gene functional differentiation. For example, the SbGATAs genes within the same subfamily show a similar distribution of introns and exons, whereas genes from different subfamilies show significant differences. The association between structure and function provides key clues for in-depth research into the specific roles of the SbGATAs genes in growth, development, and stress responses.

### 4.2. Mining of Potential Genes Regulating Chlorophyll Biosynthesis in the GATA Gene Family of S. baicalensis

Studies have shown that GATA transcription factors can promote the development of plant chloroplasts and the formation of chlorophyll. In *A. thaliana*, *GNC* (GATA Nitrate-inducible Carbon-metabolism-involved) and *GNL*/*CGA1* (GNC-Like/Cytokinin-responsive GATA1) are two major transcription factors in the chlorophyll biosynthesis process, which mediate the conversion of plastids to chloroplasts by acting on CK and promote the development of chlorophyll [50,51]. The expression of *GNC* and *GNL*/*CGA1* is also affected by gibberellin and auxin through cross-regulation at the transcriptional level to influence the development of chloroplasts and the synthesis of chlorophyll [52,53]. In *O. sativa*, *Cga1* (CYTOKININ-RESPONSIVE GATA TRANSCRIPTION FACTOR1) (*OsGATA11*, *LOC_Os02g12790*) plays an important role in regulating plant morphology and chloroplast development [54]. This gene shows increased expression in response to light, nitrogen, and cytokinin treatments, while it is reduced in the dark and by gibberellin. Through overexpression and RNAi silencing experiments, *OsGATA12* (*LOC_Os03g61570*) regulates chloroplast development and plant senescence [55]. *OsGATA16* (*LOC_Os06g37450*) acts as a positive regulatory factor controlling chlorophyll biosynthesis and chloroplast development [56].

*AtGNC*, *AtGNL*/*CGA1*, *OsCga1*, *OsGATA12*, and *OsGATA16* all belong to the Class B subfamily. Using phylogenetic analysis, five genes closely related to the same subfamily were selected for expression pattern analysis: *SbGATA6*, *SbGATA8*, *SbGATA14*, *SbGATA18*, and *SbGATA24*. *SbGATA8* is more closely related to *OsCga1*, *OsGATA16*, and *AtGNC*; *SbGATA24* and *OsGATA12* are more closely related.

### 4.3. Analysis of Chlorophyll Content Changes in Leaves After Foliar Application of Carbon Dot Materials and the Potential Role of the SbGATA Gene

This study employed RT-qPCR to elucidate the expression patterns of the *SbGATAs* family members in *S. baicalensis* leaves after foliar application of carbon dots at different concentrations. The results indicated that, at low concentrations, except for *SbGATA6*, which was upregulated 2.1-fold, the other four genes were downregulated. At high concentrations, except for *SbGATA6*, which was upregulated by 3.75-fold, and *SbGATA14*, whose expression level was nearly the same as that of the control, the other three genes were all downregulated. Notably, *SbGATA6* was the only gene significantly upregulated at both concentrations, and its upregulation fold increased with concentration, suggesting that it may exhibit a broad response to carbon dot treatment and may be involved in physiological regulation at different concentrations. Members of the GATA transcription factor family have complex mutual regulations. High-concentration carbon dots may directly promote photosynthesis through their photoconversion properties, thereby driving the net increase in chlorophyll content; while *SbGATA6* may act as a multifunctional carbon dot response factor, simultaneously participating in stress adaptation and photosynthesis-related processes. However, its specific function and causal relationship with chlorophyll synthesis still need further verification.

## 5. Conclusions

Based on genomic data from *S. baicalensis*, this study identified 25 members of the *SbGATA* gene family and conducted a comprehensive analysis of their physicochemical properties, subcellular localization predictions, gene structures, phylogenetic relationships, cis-regulatory elements, and expression patterns following leaf surface application of carbon dots. The study also analyzed their evolutionary characteristics and potential regulatory pathways. These proteins showed significant differences in physicochemical properties, yet they were all hydrophilic. The subcellular localization predictions were all located in the nuclear region. The members of this gene family were unevenly distributed across the seven chromosomes of *S. baicalensis*, and nine pairs of *GATA* genes underwent segmental duplication. These gene promoters contained a large number of cis-regulatory elements, including those responsive to light, hormones, and stress, indicating that these GATA proteins may be involved in various biological processes, such as growth and development, and responses to biotic and abiotic stresses, in *S. baicalensis*. At the same time, this study screened key *GATA* genes that responded significantly to carbon dot induction based on expression patterns, combined with cis-elements and functional annotations, to predict their potential functions in growth and development, stress resistance, and regulation of secondary metabolism, thereby providing candidate genes for subsequent gene function verification.

## Figures and Tables

**Table 1:** Basic information of members of the *SbGATA* gene family.

Gene Name	Sequence ID	Chromosome	Number of Amino Acid	Molecular Weight	Theoretical pI	Instability Index	Aliphatic Index	Grand Average of Hydropathicity	Subcellular Localization Predicted
SbGATA1	Sb01t21140.mRNA1	Chr1	318	34.74503	6.01	57.71	61.01	−0.594	Nucleus.
SbGATA2	Sb01t29680.mRNA1	Chr1	307	33.43655	6.00	57.34	64.53	−0.554	Nucleus.
SbGATA3	Sb01t35710.mRNA1	Chr1	243	27.795	6.02	49.79	50.95	−1.012	Nucleus.
SbGATA4	Sb01t44110.mRNA1	Chr1	387	41.78982	6.24	46.05	58.55	−0.519	Nucleus.
SbGATA5	Sb01t47440.mRNA1	Chr1	342	37.21652	4.87	60.26	66.70	−0.560	Nucleus.
SbGATA6	Sb02t00900.mRNA1	Chr2	193	21.36674	8.97	50.93	47.67	−0.794	Nucleus.
SbGATA7	Sb02t07600.mRNA1	Chr2	298	32.467	5.17	58.68	57.28	−0.685	Nucleus.
SbGATA8	Sb02t14590.mRNA1	Chr2	292	32.12819	9.50	49.14	59.49	−0.756	Nucleus.
SbGATA9	Sb02t24870.mRNA1	Chr2	303	32.90034	6.35	40.45	62.15	−0.763	Nucleus.
SbGATA10	Sb02t27310.mRNA1	Chr2	321	35.35705	5.94	46.58	58.63	−0.610	Nucleus.
SbGATA11	Sb03t03730.mRNA1	Chr3	161	18.15313	6.06	48.47	53.35	−1.245	Nucleus.
SbGATA12	Sb03t03760.mRNA1	Chr3	109	12.55884	4.99	47.10	48.17	−1.164	Nucleus.
SbGATA13	Sb03t08470.mRNA1	Chr3	329	36.39221	9.10	69.60	66.66	−0.579	Nucleus.
SbGATA14	Sb03t12230.mRNA1	Chr3	131	14.86334	10.17	76.69	69.24	−0.786	Nucleus.
SbGATA15	Sb03t13340.mRNA1	Chr3	533	59.59296	6.21	51.97	69.32	−0.577	Nucleus.
SbGATA16	Sb03t20790.mRNA1	Chr3	341	38.04143	6.23	54.17	54.69	−0.772	Nucleus.
SbGATA17	Sb03t21830.mRNA1	Chr3	245	26.71858	5.82	56.45	59.43	−0.516	Nucleus.
SbGATA18	Sb03t25200.mRNA1	Chr3	101	11.60261	10.38	74.60	55.05	−0.997	Nucleus.
SbGATA19	Sb03t27530.mRNA1	Chr3	214	24.455	9.73	45.06	67.85	−0.599	Nucleus.
SbGATA20	Sb03t28490.mRNA1	Chr3	323	35.2724	7.5	58.55	53.13	−0.571	Nucleus.
SbGATA21	Sb03t31970.mRNA1	Chr3	302	32.94261	6.22	49.39	50.40	−0.862	Nucleus.
SbGATA22	Sb04t24570.mRNA1	Chr4	339	37.3198	6.18	56.89	58.73	−0.599	Nucleus.
SbGATA23	Sb06t05030.mRNA1	Chr6	284	30.58231	6.6	54.55	65.6	−0.367	Nucleus.
SbGATA24	Sb08t12660.mRNA1	Chr8	161	17.43454	9.78	58.93	45.47	−0.951	Nucleus.
SbGATA25	Sb09t21390.mRNA1	Chr9	651	72.48420	6.01	52.48	98.40	−0.237	Cytoplasm. Nucleus.

## Data Availability

All data prepared during the research work is provided in the main body of this published article. For publicly archived datasets, hyperlinks are provided in this manuscript to access already published data used in this article.
